# The history of obstetric nurses’ professional training of in Minas Gerais (1957-1999): a genealogical analysis

**DOI:** 10.1590/0034-7167-2022-0459

**Published:** 2023-05-12

**Authors:** Rafaela Siqueira Costa Schreck, Kênia Lara da Silva

**Affiliations:** I Universidade Federal de Minas Gerais. Belo Horizonte, Minas Gerais, Brazil

**Keywords:** Obstetric Nursing, Nurse Midwives, Teaching, History, History of Nursing., Enfermería Obstétrica, Enfermeras Obstetrices, Enseñanza, Historia, Historia de la Enfermería., Enfermagem Obstétrica, Enfermeiras Obstétricas, Ensino, História, História da Enfermagem.

## Abstract

**Objectives:**

to analyze obstetric nurses’ professional training in Minas Gerais between 1957 and 1999, according to genealogical principles.

**Methods:**

a qualitative interpretative study based on historical research with genealogical analysis. Data were obtained through documentary research and oral history, with six participants, and submitted to discourse analysis.

**Results:**

they recompose the genealogical path of obstetric nurses’ professional training from Minas. The speeches reveal field of practice deprivation in professional training and the importance of the articulation between the Universidade Federal de Minas Gerais Nursing School and Hospital Sofia Feldman for teaching and work in obstetric nursing. It was identified that training, in the national scenario, evolved from a Escola de Enfermagem Carlos Chagas’ peripheral initiative to centrality and capillarity.

**Final Considerations:**

the unique historical trajectory of obstetric nurses’ professional training in Minas Gerais, marked by ruptures, institutional articulations, conflicting games and interest, was unveiled.

## RESUMO

**Objetivos:**

analisar a formação profissional de enfermeiras obstétricas, em Minas Gerais, entre 1957 e 1999, segundo os princípios genealógicos.

**Métodos:**

estudo qualitativo interpretativo, fundamentado na pesquisa histórica com análise genealógica. Os dados foram obtidos por pesquisa documental e história oral, com seis participantes, e submetidos à análise do discurso.

**Resultados:**

recompõem o percurso genealógico da formação profissional das enfermeiras obstétricas mineiras. Os discursos revelam privação do campo de atuação prática na formação das profissionais e importância da articulação da Escola de Enfermagem da Universidade Federal de Minas Gerais e do Hospital Sofia Feldman para ensino e atuação da enfermagem obstétrica. Identificou-se que a formação, no cenário nacional, evoluiu de uma iniciativa periférica da Escola de Enfermagem Carlos Chagas para a centralidade e capilaridade.

**Considerações Finais:**

desvelou-se a trajetória histórica singular da formação profissional de enfermeiras obstétricas, em Minas Gerais, marcada por rupturas, articulações institucionais, jogos de enfrentamento e interesse.

## RESUMEN

**Objetivos:**

analizar la formación profesional de las comadronas en Minas Gerais entre 1957 y 1999, según principios genealógicos.

**Métodos:**

estudio interpretativo cualitativo basado en investigación histórica con análisis genealógico. Los datos fueron obtenidos a través de investigación documental y de historia oral, con seis participantes, y sometidos a análisis de discurso.

**Resultados:**

recomponen el camino genealógico de la formación profesional de enfermeros obstetras de Minas Gerais. Los discursos revelan la privación del campo de acción práctica en la formación de profesionales y la importancia de la articulación entre la Escuela de Enfermería de la Universidade Federal de Minas Gerais y el Hospital Sofia Feldman para la enseñanza y actuación en enfermería obstétrica. Se identificó que la formación, en el escenario nacional, evolucionó de una iniciativa periférica de la Escola de Enfermagem Carlos Chagas a la centralidad y capilaridad.

**Consideraciones Finales:**

se develó la trayectoria histórica única de la formación profesional de enfermeras obstétricas en Minas Gerais, marcada por rupturas, articulaciones institucionales, juegos de confrontación e interés.

## INTRODUCTION

Nursing education regulation in Brazil occurred at the end of the 19^th^ century. Since that period, the profession has gained space and respect in the field of health, especially with regard to women’s health^(1-2)^.

With regard to midwifery, since 1832, lay midwives, who worked in woman care during childbirth, began to receive formal education in medical schools, classified as midwife, specialized nurse, obstetrician and obstetric nurse. After the enactment of Law 775/1949, federal or recognized nursing schools were authorized to create specialization courses in midwifery nursing after graduation in general nursing. As a result, two distinct professional categories were created: obstetric nurses, formed by nursing schools, and midwives, formed by medical schools. This legislative change started a conflict between the categories of midwives and obstetric nurses, who differed in relation to the limits and attributions of exercising each profession^(1)^.

In 1955, Law 2604 was enacted, which regulated nursing practice at the same time that it differentiated obstetric nursing professionals, assigning them exclusive activities^(3-4)^. In the 1960s, the Federal Council of Education, through Opinion CFE 271/62, set the minimum curriculum for the nursing course lasting three years. In this curriculum model, training took place over a common core of two years and a diversified, optional year, characterized as specialization. At the same time, midwives and specialists in midwifery continued to be trained^(1,5)^.

In the 1970s, after implementing the university reform, which began in 1968, the undergraduate nursing course’s minimum curriculum was restructured, by Opinion 163/72 of the Federal Council of Education^(6)^, and midwifery courses were transformed into qualifications, in undergraduate courses, under the responsibility of nursing schools. The nursing curriculum began to consist of three phases: pre-professional, common professional core and qualifications in public health, medical-surgical nursing or midwifery, to be taken optionally. At that time, nursing schools graduated students in nursing and midwifery courses, regardless of compliance with the specific content in midwifery^(5,7)^.

A new change in the nursing curriculum took place in 1994, under Opinion 314/94, with the extinction of qualifications and general training for nurses, shifting specific training, including midwifery, to graduate courses in the *lato sensu* modality^(8)^.

As of 1999, with a view to improving obstetric and perinatal care quality, the Ministry of Health invested in obstetric nurses’ training through agreements signed with universities across the country, and funding of specialization courses as a way of expanding the number of these professionals in the health system. This initiative became a national symbol in the project to train nurses in maternal and perinatal care^(9)^.

In Minas Gerais, over the years, obstetric nurses’ training process has achieved notoriety with tradition in training obstetric nurses. The *Universidade Federal de Minas Gerais* Nursing School (EEUFMG) plays an important role in the history of midwifery nursing in the state, with a broad trajectory in practical and theoretical teaching of midwifery^(2,10)^. In this trajectory, obstetric nurses’ professional training in Minas Gerais went through several stages, and it is assumed that it was marked by rupture and continuity, evolving from peripheral training, with institutional initiative, to centrality and capillarity with models of specialization and multidisciplinary residency, guided by the encouragement of public policies.

Given this context, it is necessary to disseminate and analyze the professional training process of obstetric nursing in Minas Gerais, mainly, advancing in the understanding that all knowledge is a historical construction, formed from real games, and that it is connected with social circumstances, behaviors, decisions and disputes^(11)^.

## OBJECTIVES

To analyze obstetric nurses’ professional training, in Minas Gerais, between 1957 and 1999, according to genealogical principles.

## METHODS

### Ethical aspects

The documents that are used in the research are in the public domain. For the interview phase, the research followed the considerations of Resolutions 466/2012 and 510/2016, with approval by the Research Committee Ethics. Participants are coded to ensure confidentiality and anonymity.

### Theoretical-methodological framework

The study is anchored in Michael Foucault’s philosophical conceptions, specifically genealogy, which allows revealing conditions of possibility, events, conflicting games, ruptures and legitimization of certain discourses in the formation of knowledge, subjects and obstetric nursing practice in Minas Gerais.

To compose this study, according to Michael Foucault’s principles, we sought the effective history of the research object, genealogically directed, which distances itself from the linearity of time, teleology, objectivity and accuracy of facts, seeking discontinuity, relationships, subjections, coping and struggles in the interpretation of events^(12)^.

### Study design

This is an interpretative qualitative study based on historical research. The criteria proposed by the COnsolidated criteria for REporting Qualitative research (COREQ) were used.

### Methodological procedures

The study is part of a doctoral thesis, in which documentary research was used, due to the availability of written sources, and oral history, and the presence of important subjects who lived and experienced the beginning of obstetric nursing training process in Minas Gerais. The criterion for selecting documents was the mention or free speech about the first specialization courses in obstetric nursing, offered by *Escola de Enfermagem Carlos Chagas* (EECC), current EEUFMG. Regarding oral sources, the inclusion criterion was the identification, based on the survey of written documents, of the key names of subjects directly or indirectly involved with these courses.

### Study setting

The study was conducted in the state of Minas Gerais, whose data referring to training of 1957 and 1999 were analyzed. The year 1957 marks the first events for obstetric nurses’ professional training from Minas Gerais. The study covered documentary collection and interviews, which took place on-site, according to participants’ and researchers’ availability.

### Data source

Data were obtained from documents from collections of the Memory Center of the Nursing School (Cemenf - *Centro de Memória da Escola de Enfermagem*) and the Memory Center of the Faculty of Medicine (Cememor - *Centro de Memória da Faculdade de Medicina*) of the *Universidade Federal de Minas Gerais* (UFMG), as shown in Charts 1 and 2. Interviews were carried out in person, with six obstetric nurses, professors and former professors at the EEUFMG, who participated in the organization and offering of the 1999 Obstetric Nursing Specialization Course, which marked the resumption of training for obstetric nurses in Minas Gerais, after thirty-three years without specialist training.

**Chart 1:** Documents from the physical and digital collection of Memory Center of the Nursing School (*Centro de Memória da Escola de Enfermagem*) and Memory Center of the Faculty of Medicine (*Centro de Memória da Faculdade de Medicina*) consulted

Document	Date	Topic
D1 - EECC graduate registration in obstetric nursing.	1957	List of registered students.
D2 - Minutes of the 6^th^ Session of the Faculty of Medicine Congregation.	March 26, 1958	Opinion of the Faculty of Medicine Teaching Commission on the completion of a specialization course for obstetric nurses at the EECC.
D3 - Internal regulation of the Specialization Course in Obstetric Nursing at EECC.	1966	Purpose, objectives and curricular structure of the 1966 Specialization Course in Obstetric Nursing.
D4 - Minutes of the EECC faculty congregation.	1966	Minutes of the congregation dealing with the creation of the 1966 obstetric nursing specialization.
D5 - Preliminary draft of the Qualification Course in Midwifery or Midwifery at the EEUFMG.	1979	Description of the objectives and curricular outline of the draft qualification in obstetric nursing.
D6 - Opinion of the Graduate Council 070/80 - EEUFMG.	1980	Opinion issued on the creation of qualification in obstetric nursing.
D7 - Project to Create the Specialization Course in Obstetric Nursing.	1997	Curriculum structure presented to the Department of Maternal-Infant Nursing and Public Health at the EEUFMG.
D8 - Opinion of the UFMG Graduate Chamber CPG/UFMG/004/98.	1998	Favorable opinion on the creation of the 1999 Obstetric Nursing Specialization Course.

*EECC - Escola de Enfermagem Carlos Chagas; EEUFMG - Universidade Federal de Minas Gerais Nursing School.*

**Chart 2:** Documents from Memory Center of the Nursing School’s (*Centro de Memória da Escola de Enfermagem*) oral collection consulted

Interview transcribed	Biography
D9 - Carmelita Pinto Rabelo	Director of EECC in 1967.
D10- Aparecida Ferreira Moura	Obstetric nursing specialization professor at the EECC in 1957.

*EECC - Escola de Enfermagem Carlos Chagas.*

### Data collection and organization

Collection was carried out from August 2021 to February 2022. For each document, a reading form was created containing the summary, bibliographic reference and transcription of all important excerpts for analysis. The interviews were conducted using a semi-structured script with 5 guiding questions about participants’ memories of their midwifery training in Minas Gerais. The interviews were recorded in audio and later transcribed, with an average duration of 1 hour and 48 minutes, with a total of 4 hours, 22 minutes and 55 seconds.

### Data analysis

The documents and interviews used as a research source were submitted to discourse analysis (DA), explained by Michael Foucault. The choice for this mode of analysis was due to the relationship with the theoretical framework adopted and its ontology of critical and emancipatory stance that seeks to go beyond the understanding of discourse as a set of signs, as significant elements that refer to certain contents or representations.

In view of these DA principles and having Foucault’s genealogy framework as a guiding principle, the stages proposed for the analysis process of this study were influenced by Carabine^(13)^ and Prado Filho^(14)^, consisting of two steps: source selection and data knowledge; and genealogical discourse analysis.

Genealogical discourse analysis was divided into themes and objects (events; contexts/social, formative, political and legislative landmarks) and strategies and techniques (discursive elements: narrative construction; choices for certain lexical terms that allowed identifying resistances, disputes, strategic games, flows and movements of power relations).

Two analytical categories emerged: *Field of practice deprivation as continuity in the training and work of obstetric nurses*; and *Articulation between the Universidade Federal de Minas Gerais Nursing School and Hospital Sofia Feldman: a new order in training and work of obstetric nursing*.

## RESULTS

### The first training initiatives for obstetric nurses in Minas Gerais: specializations in 1957 and 1966

The first initiatives of specialization courses in obstetric nursing, in Minas Gerais, took place in 1957 and 1966 by EECC, current EEUFMG. The discovery of the existence of the first specialization course, in 1957, entitled “Graduate Course in Obstetric Nursing”, took place after a detailed search in Cemenf’s collection. The first clues came from the institution’s enrollment book, which contained, on the final pages, the registration of nine students in the course (D1).

The course lasted one year, with enrollments recorded only for the first course, which began in 1957. However, in the interviews transcribed from oral collection, two groups are mentioned, which may have trained a total of twelve students, nine of whom were from the first class. These students came from different parts of the country, including the states of Ceará, Bahia and São Paulo.

At the EECC, for creating and implementing the course, a necessary flow, for the emergence of this event, was the interlocution with midwifery professors at the UFMG Faculty of Medicine. Nursing professors were responsible for the course’s theoretical and practical content and taught the so-called “basic subjects”, which were taught by medical professors. The course’s curricular content was structured in the following subjects: Anatomy and Specialized Physiology; Gynecology; Histology; Anesthesiology; Hematology; Home Midwifery; Physiological Midwifery and Prenatal Hygiene; Prenatal Care; Medico-Social Obstetric Assistance; Normal and Pathological Midwifery; Neonatal Childcare; Deontology; Nursing in Gynecology and Hospital Nursing (D1).

Conflicting games between medicine and nursing for obstetric nurses’ training are expressive in the refusal of the new direction of *Hospital das Clínicas* to offer the second group of the specialization course. Professor Aparecida Ferreira relates the end of the course to change in management at the Faculty of Medicine, describing the succession of the position of clinical director of midwifery as “authoritarian, very impolite”:

*It’s because, in 1957, when* [clinical director], *he left the head of midwifery. What he assumed was very authoritative, very impolite, he yelled and sometimes he didn’t want the nurses to deliver. We said it was a specialization course, it showed the law, but for him, it didn’t matter.* (D10)

The Minutes of the Faculty of Medicine Congregation, regarding the conduction of this specialization course in obstetric nursing, reveals the broad discussions and strategies undertaken by medical professors to limit the role of nurses to prenatal and postpartum care and guarantee “entirely the births for the learning of future physicians”.

*Regarding the realization of the 2^nd^ specialization course for obstetric nurses at the EECC, the following opinion was issued, “Ask Prof. Daniel who seeks to help the graduation course of future midwives, perhaps paying attention to the circumstance that the essential objective of teaching for nurses is preand post-natal care, leaving entirely the deliveries for future physicians to learn. After the Opinion was widely debated, the matter was forwarded to the Teaching Commission for further study* [...]. (D2)

The offer of, perhaps, two classes of specialization in midwifery nursing, took place with professors who are specialists in the area and a curriculum characterized by knowledge specific to nursing. However, despite these conditions, the closure of internship field at *Hospital das Clínicas*, where the course’s practical part took place, was decisive for the interruption of obstetric nurses’ training in Minas Gerais during this period.

The training of nurses with the title of specialists in midwifery took place again, at the EECC, in 1966, with the offer of a new class of the Specialization Course in Obstetric Nursing (D3). According to the minutes of the congregation of EECC professors, the course vacancies were limited, being destined only for students who graduated in 1965. For planning and collaboration in the course, three nursing professors were hired during this period: Maria Noemi Ribeiro, Luzia da Silva and Inês Lemos da Fonseca (D4).

The 1966 Graduate Course in Midwifery was aimed at improving nurses, being divided into two periods of 5 months each. The curriculum included Nursing in Gynecology and Midwifery, Nursing in Public Health and Psychiatry integrated with Obstetric Nursing. Among the complementary subjects were the basic cycle subjects and the so-called Social Midwifery, which was intended for caring for women in conditions of poverty and vulnerability, such as “abandoned mothers”. (D3). The objectives described in the course’s internal regulation have important characteristics, which may indicate an innovation in the profession field design, with an interest in improving the professional class:

*Objectives: 1- Give nurses the opportunity to acquire more technical-scientific knowledge, aiming at improving the group and the class. 2 - Contribute to the increase of personnel capable of providing supervision and care for maternity and childhood. 3 - Awaken the interest of nurses in contributing to the reduction of local statistics with regard to maternal and child morbidity and mortality.* (D3)

Records about this specialization are scarce, with evidence of offering only one group and training four specialist nurses. The course is not offered again, marking a break in the training process of obstetric nurses from Minas Gerais.

### Absence of qualification in midwifery: from 1972 to 1994

Later, in 1972, with Opinion 163/1972 of the Federal Council of Education, the EEUFMG, which had achieved, in 1968, the status of an autonomous unit integrated to UFMG, being renamed, did not offer the qualification in midwifery. Despite the elaboration of a preliminary project for this qualification, aiming at “preparing obstetric nurses with solid knowledge, skills and attitudes for a safe assistance for mothers and children, during the pregnancy-puerperal cycle”, there was a defined curricular structure (D5).

The reasons for refusal to conduct qualification in midwifery were lack of teaching resources and fields of practice for training students, added to financial crisis faced by the university (D6). The interviewees’ reports and oral documents confirm the impossibility of carrying out qualification in midwifery, at the EEUFMG, with regularity in speeches about the limiting factors for training qualified obstetric nurses:

[...] *it was very difficult for three people to sustain a qualification in midwifery.* (D9)

[...] *but you also didn’t have the scenario to continue with training, right? About obstetric nurses* [...] *I’m talking here at school, it did not exist!* (E3)

*That is the history of obstetric nursing, it is: when childbirth was institutionalized, the doctor took over, and nurses, midwives lost their space* [...] *then, schools also stopped training! But here at school, you also never had a qualification in midwifery.* (E4)

Only in 1999, with the validity of Opinion 314/94 of the Federal Council of Education, specialization in obstetric nursing was offered again by EEUFMG. The interviewees cite the increase in the number of surgical deliveries, lack of professors and field of training deprivation and work associated with disputes and hegemony of medical practice as motivating factors for this interruption of nursing qualification in Minas Gerais.

*Because we were at the PEAK of cesarean sections* [...] *at the PEAK of interventions! The doctor didn’t want to KNOW about NURSES!* (E1)

[...] *not only Belo Horizonte, right? UFMG* [...] *but the whole of Brazil stopped training because obstetric nurses didn’t have space to work! And the AREA is not a simple area to work on, right? It is an area of great conflict to act, BETWEEN categories* [...] *so, it was a DARK time for obstetric nursing, right? Because, how would you TRAIN if you didn’t have a practice field, right?* (E4)

*It’s always in the field of practice, right?* [...] *that’s the difficulty! And we always come up against this medical power, right?* [...] *all those disputes! So, they didn’t want to train people other than physicians at the hospital, right?* (E5)

The discursive elements used by participants and outlined above express the childbirth assistance scenario in this period that determined the suspension of training of specialist nurses in midwifery in Minas Gerais: “peak of the interventions”, “they had no space to work”, “a dark time for obstetric nursing”. The medical class’s stance and interests in dominating the obstetric field and the dispute relations that led to deprivation of nursing activities, with impacts on the interruption of professional training, are also expressed.

These conditions that led to deprivation of professional training field did not mark the end of a flow, but awakened to other searches in the genealogical construction of obstetric nursing in Minas Gerais.

### Resumption of training for obstetric nurses: specialization in 1999

The next offer of a specialization course in obstetric nursing, in Minas Gerais, took place in 1999, from articulation of two institutions: EEUFMG and *Hospital Sofia Feldman* (HSF). The proposal for this specialization arises from the need to meet legal regulations and workforce qualification for nurses’ practice in childbirth care in the state. To this end, there was an agreement between teaching and practice with the EEUFMG faculty, PhD, master’s and specialist professors in midwifery, trained by *Escola Paulista de Enfermagem*, and the field of practice of HSF (D8).

The interviews’ speeches reveal the negotiations, interests and relationships involved in offering a new Specialization Course in Obstetric Nursing, in the late 1990s, fruit of the consolidation of this articulation between EEUFMG and HSF professors, institutions represented in the speeches by the clinical director and by a retired professor from the school who worked in teaching and research at the hospital.

*Then the* [director] *said, “Let’s do a specialization course!”* [...] *the train went like this: pa-pa-pa* [speed] [...] *there, that’s why I say that the specialization was born, first, because of having Sofia* [...]. (E2)

*Then, we were attracting people, since Sofia was also willing to be in the field* [...] *the* [director] *also did the negotiation, right? He inserted the field of obstetric activity to nurses, the school did the theoretical teaching part* [...] *and Sofia, the practical part, and did this intermediation.* (E3)

*It was a need in the field, because Sofia needed qualified labor, right? And the UFMG Nursing School also had a chart of professors for this, right? Sofia had a need, and the school had the potential to graduate and awarded the title!* [...] *the other important link, which I believe was the* [nurse-professor], *because she was a professor at the school and was Sofia’s at the time, right? So, she knew that and she was also a facilitator for it to happen, you know?* (E4)

The consulted documents show that, although the first class was offered in 1999, the project for the Specialization Course in Obstetric Nursing has been filed at UFMG since 1997, with a proposal for training in the residency modality, with a workload of 3,390 hours (D7).

Later, throughout the state of Minas Gerais, there was an expansion of the professional training of specialists in obstetric nursing, with the emergence of other institutions and training centers. At the EEUFMG, between 1999 and 2012, 14 courses were offered with the training of 230 obstetric nurses. In 2013, residency training began to be offered, with funding from the Ministry of Health, in a strategic action to qualify professionals to improve the indicators of woman care.

This expansion of obstetric nurses’ training from Minas Gerais was also expressed in interviewees’ speeches, when they presented the national impact of HSF and EEUFMG for the teaching and insertion of these professionals. In this regard, using expressions such as: “the thing took shape”, “training took off”, “introduction of midwifery in the state and in Brazil” and “expanded to the state, to Brazil, to the world”.

*This whole experience that the school had in training obstetric nurses was a very important prerequisite to later increase training, and the thing took shape* [...] *I think we had a very fast advance, from the point of view of history* [...] *training took off!* (E3)

*This performance by Sofia, of course, is fundamental for the introduction of midwifery in the state and in Brazil, certainly, for humanized care.* (E5)

*But Sofia’s growth, right? It expanded to the state, to Brazil, to the world* [...] *we have advanced a lot in Minas Gerais. TODAY, we are recognized, right?* (E6)

## DISCUSSION

### Deprivation of field of practical action as continuity in obstetric nurses’ training and work

Michel Foucault’s work on genealogy analyzes how events arise at specific historical points and can then be interpreted as natural or normalized events. For the author, the events are understood by the multiplicity of forces present in a historical process that allow the understanding of a social phenomenon^(12,14)^.

In this sense, the first initiatives for obstetric nurses’ training by the EECC, in 1957 and 1966, and the discussions on qualification in midwifery characterize important events in the construction of obstetric nursing in Minas Gerais. The speeches’ singularities and rarities recovered about these courses give visibility to games of interest and equations of forces, between medicine and nursing, involved in the training and performance of the first obstetric nurses in the state.

The analysis proposed in this study also starts from understanding the profession as an occupation that presents a structured body of complex theoretical and practical knowledge, which is transmitted in the course of a long process of learning and training and which establishes the authority of professionals vis-à-vis lay people and their autonomy^(15)^.

For care practice, nursing professionals need, during their training, learning that brings theory closer to practice, developing a practice based on knowledge. Know-how constitutes a tool for nursing, based on skills and knowledge in the exercise of care^(16)^.

Thus, from the moment the first specialization courses are offered, even though linked to the Faculty of Medicine, a movement begins to build the professional field of obstetric nursing in Minas Gerais, with its own body of knowledge and human resources that gives visibility to obstetric nurses’ practice in childbirth care.

This body of knowledge is evidenced, in specialization courses of 1957 and 1966, by the construction of a curriculum grid with specific subjects for the know-how of obstetric nursing, which began to characterize it as a profession and mark its emergence in the scenario obstetric. It should be noted that, at that time, with the increasing childbirth hospitalization, there was a national movement to consolidate obstetric nurses’ practice, who still competed with midwives for the space of care. Therefore, specialization courses in obstetric nursing were strategic for preparing nurses capable of guaranteeing work in the hospital field^(1,5)^.

The documents and discourses analyzed indicate that the training and performance of the first obstetric nurses were, from the beginning, regulated by the disciplinary power of the medical category. This power, in essence relational, operated in docility-utility relations and in the normalizing sanction of training and practice regulation.

Docility-utility relations, which refer to the method of docility of bodies to control the operations of individuals, subjecting their strength to what is desired^(12)^, were built between physicians and nurses. Physicians, who since the 19^th^ century had controlled the training of midwives and obstetric nurses in the country^(1,5)^, in the 1957 specialization course, by making training possible and allowing nurses to work in hospital institutions, they also ensured, under guardianship and supervision, the production of a docile and useful workforce for the development of medicine itself.

The normalizing sanction, whose Foucault’s definition encompasses the micropenalties that qualify and repress^(17)^, regulated obstetric nurses’ training and practice, expressed in deprivation of internship fields, to follow the specialization course in 1957, as a strategic situation for limit the growth of midwifery in Minas Gerais. Furthermore, this sanction is expressed in the imposition of nurses’ teaching and work, and in the care given to women, only in the preand post-natal period, to guarantee the exclusivity of physicians in assisting childbirth.

In the national context, birth care, from the 1960s onwards, began to be characterized by the growth in the number of surgical childbirths, mainly with the growing expansion of hospital care^(18)^.

Subsequently, with the dominance of medical practice, the growing institutionalization of birth and, consequently, the increase in the number of cesarean sections, practice field deprivation was established as a continuity in discourses for the limitation of professional training in obstetric nursing. Likewise, the lack of fields for practice, with the increase in the medicalization of birth, and the demand for courses and trained professors were the main reasons for the interruption of specialization courses in other parts of the country^(10,19)^.

### Articulation between the *Universidade Federal de Minas Gerais* Nursing School and *Hospital Sofia Feldman*: a new order in training and work of obstetric nursing

In Brazil, the movement for the humanization of birth was driven throughout the 1970s and 1980s, based on professionals’ experience with midwives’ traditional practices, indigenous peoples and alternative therapy groups. This movement, along with advances in the organization of perinatal and neonatal care in the country, sought to establish birth care practices centered on women’s autonomy and the physiology of birth^(18,20)^.

In this context of the discourses that began to circulate nationally about possibilities for changes in childbirth care, in the genealogical path of this research, in 1982, in Belo Horizonte, the inauguration of HSF stands out. The history of creation of this hospital is peculiar, the result of articulation of health professionals, philanthropy by the São Vicente de Paulo Society and community members from the North region of the city, whose management and organization of care were based on meeting the population’s health needs^(21)^.

In the process of searching for events that marked the construction of obstetric nursing in Minas Gerais, in the 1980s, the articulation between EEUFMG and the newly created HSF was revealed. This hospital assumed a counter-conduct space in the obstetric field, by establishing the logic of an assistance offered, mainly by nursing. It is emphasized that counter-conduct can be understood as a different attitude in relation to the instituted and normalized modes^(22)^.

In genealogical analysis, Foucault, from a strategic formulation to look, study, write and act in relation to historical events, explores the power within a microphysics, a web of relationships, which are exercised with a view to targets and objectives^(12,14)^. Thus, from this perspective, the interviews’ speeches reveal the composition of a web of relationships between the 1980s and 1990s, which were fundamental to increase the visibility and position obstetric nursing in facing the then medical model, centered on childbirth care, in Minas Gerais. This web is composed from the meeting of HSF health professionals and professors from the UFMG Nursing School, aligned in the same proposal of meeting women’s health needs and in defense of knowledge of humanization of childbirth.

In the development of the process of humanization of birth and childbirth in Brazil, 1998 stands out, with important actions by the Ministry of Health to regulate obstetric nurses’ practice in the country. In that year, Ordinance 2815 of May 29, 1998, which proposed assistance to low-risk normal births by obstetric nursing professionals, and Ordinance 163 of September 22, 1998, which granted obstetric nurses the possibility of issuing a Hospital Admission Authorization and including this professional in the Unified Health System’s payroll, were published^(23)^.

In this regard, the eruption of these events, such as the EEUFMG and HSF articulation, the possibility of nurses acting in birth care in the legal scope and in the HSF practice, national movement for the humanization of birth and the change in legislation for *lato sensu* nursing training, from 1994 onwards, formed a new order for obstetric nursing in Minas Gerais.

From these events, games of interests and web of relationships, there is a shaping of obstetric nursing in Minas Gerais, with the appearance of a transforming discourse that established the availability of a field of practice, bringing the materiality of professional training with the specialization course in 1999.

Thus, in Minas Gerais, the dissolution of a discourse of impossibility of training and acting in obstetric nursing began, due to deprivation of field of practice, inserting a new order in the historical trajectory of this category, with the possibility of training and acting practice. Since then, Minas Gerais, in the training process of obstetric nurses, has evolved from EECC’s institutional initiative to a rise in the national context, being among the main states in the country with the highest number of specialization and residency courses.

Moreover, Minas Gerais educational institutions participated in proposing public policies in the area of women’s health, conducting research on good practices in childbirth care and coordinating Ministry of Health initiatives for changes in training, care and management models, such as the Apice On Project (Improvement and Innovation in Care and Teaching in Midwifery and Neonatology - *Aprimoramento e Inovação no Cuidado e Ensino em Obstetrícia e Neonatologia*)^(24)^.

#### Study limitations

The limitations of this study are related to the limited number of interviews carried out, which may have affected gathering the investigated data. However, it is noteworthy that all available subjects, who experienced the historical facts of this study’s theme, were included in the research. Another limitation concerns the difficulty in surveying documents about the education of obstetric nurses, but even so, it was possible to delineate the historical construction of professional trainin, in Minas Gerais, within the time frame analyzed.

#### Contributions to health, nursing or public policy

This study was relevant, as it enabled the understanding of the teaching process and practical work of the first obstetric nurses from Minas Gerais. The genealogical approach proved to be a critical tool capable of finding the singularity and proliferation of events that marked the formation of obstetric nursing and the points of insurgency in relationships that sustain the professional routine of this category.

### FINAL CONSIDERATIONS

Obstetric nurses’ professional training, in Minas Gerais, has a unique historical trajectory marked by ruptures, institutional articulations, conflicting games and interests. The discourse of field of practice deprivation, with the impossibility of acting, determined the interruption of the first initiatives of specialization courses and the absence of qualification, demonstrating the centrality of practical teaching in obstetric nurses’ training.

From the 1990s, with the growth of the humanization movement, changes in legislation and the availability of HSF practice space, the field of Minas Gerais obstetric nursing was formed. Since then, with the conditions of possibility for teaching and practical action, obstetric nurses’ training, in Minas Gerais, acquired centrality and was able to raise a capillary network of teaching for the category, including participation in the proposition of national policies for professional training.

It is believed that continuity of discussions and research on this topic is relevant, especially considering the genealogical framework and different temporal outlines, which can reaffirm and enhance the understanding of the historical construction of obstetric nursing in Minas Gerais.

## Data Availability

https://doi.org/10.48331/scielodata.U6Z6UR
